# Temperature Self-Compensated Refractive Index Sensor Based on Fiber Bragg Grating and the Ellipsoid Structure

**DOI:** 10.3390/s19235211

**Published:** 2019-11-28

**Authors:** Binbin Yan, Lei Sun, Yanhua Luo, Liwei Yang, Haifeng Qi, Xiao Chen, Kuiru Wang, Jinhui Yuan, Xinzhu Sang, Chang Wang, Pengfei Lu, Gang-Ding Peng

**Affiliations:** 1 State Key Laboratory of Information Photonics and Optical Communications, Beijing University of Posts and Telecommunications, Beijing 100876, China; sunei1346@163.com (L.S.); krwang@bupt.edu.cn (K.W.); yuanjinhui81@bupt.edu.cn (J.Y.); xzsang@bupt.edu.cn (X.S.); photon@bupt.edu.cn (P.L.); 2 Photonics & Optical Communications, School of Electrical Engineering and Telecommunications, University of New South Wales, Sydney 2052, Australia; g.peng@unsw.edu.au; 3 College of Information and Electrical Engineering, China Agricultural University, Beijing 100083, China; yangliwei@cau.edu.cn; 4 Laser Institute, QiLu University of Technology (Shandong Academy of Sciences), Jinan 250014, China; qihf@sdlaser.cn (H.Q.); wang960100@163.com (C.W.); 5 College of Science, Minzu University of China, Beijing 100081 China; xchen4399@126.com

**Keywords:** refractive index sensor, fiber Bragg grating, ellipsoid, temperature self-compensated

## Abstract

In this paper, a temperature self-compensated refractive index sensor based on fiber Bragg grating (FBG) and the ellipsoid structure is demonstrated. The ellipsoid can excite the cladding modes and recouple them into the fiber core. Two well-defined wavelength bands are observed in the reflection spectrum of the proposed sensor, i.e., the Bragg resonant peak and the cladding resonant peaks. By measuring the wavelength shift of the cladding resonant peak, the surrounding refractive index (SRI) can be determined, and the wavelength shift of the Bragg resonant peak can be used as a reliable reference to self-compensate the temperature variation (temperature sensitivity of 10.76 pm/°C). When the SRI changes from 1.3352 to 1.3722, the cladding resonant peak redshifts linearly with an average sensitivity of 352.6 pm/RIU (refractive index unit). When the SRI changes from 1.3722 to 1.4426, an exponential redshift is observed with a maximum sensitivity of 4182.2 pm/RIU. Especially, the sensing performance is not very reliant on the distance between the FBG and the ellipsoid, greatly improving the ease of the fabrication.

## 1. Introduction

The refractive index sensor has many important applications, such as the quality control of chemical industries, monitoring biochemical reactions, the prevention of global warming, etc. [1,2,3,4,5,6,7]. The most famous refractive index sensor is the Abbe refractometer, which measures the surrounding refractive index (SRI) according to the total internal reflection [8]. Although this instrument has a high accuracy, the application is greatly restricted by its large size and monitoring manner. A microresonator based on the silicon-on-insulator technology has also been used to monitor the SRI [9,10,11], but it suffers from high cost and a complicated fabrication process.

Optical fiber-based sensors have attracted much attention due to their unique advantages [12,13,14,15,16], such as compact size, immunity to electromagnetic interference, and fast response time, which are based on a variety of principles and structures, such as fiber gratings, fiber lasers, fiberoptic interferometers, etc. Therefore, they have widely been used to monitor the temperature [12], the curvature [16], the gas [15], etc. [17]. In recent years, several types of fiber refractive index sensors have also been demonstrated by monitoring the effective index changes of the guided modes, which are caused by the perturbation of the SRI [18,19,20,21,22]. A microfiber-based refractive index sensor can realize the SRI sensing with ultrahigh sensitivity; however, it suffers from extreme mechanical instability [23,24,25]. Mach–Zehnder structure-based refractive index sensors can stably be applied in practical measurements, but the sensing system is complicated [26]. The fiber grating-based refractive index sensor has many advantages, including that it has strong mechanical stability, is easy to embed, and allows the simultaneous measurement of multiple parameters [27,28,29,30,31]. In fact, for the most classic fiber grating, the fiber Bragg grating (FBG), is naturally insensitive to the SRI variation, since the reflected guided mode is mainly confined in the fiber core [17]. Compared with the confined core mode, cladding modes can strongly interact with the surrounding medium, which offers great potential for the SRI sensing. Thus, long-period fiber grating (LPFG) is proposed for the SRI sensing, because it implements light coupling between the core mode and cladding modes to provide resonant peaks in the transmission spectrum, which are easily influenced by the SRI changes [32,33]. However, the LPFG-based refractive index sensors are not compact because they can only be operated in transmission mode [34]. The combination of FBG and LPFG has further been investigated for SRI sensing [35], but it will undoubtedly increase the fabrication complexity requiring two sets of grating writing devices, and this structure is not conducive to system integration. In addition, tilted fiber Bragg grating (TFBG) is another candidate for SRI sensing, but the development is hindered by its fabrication complexity [36,37].

Here, we propose an SRI sensor based on FBG cascading the ellipsoid structure, which is simple, compact, and easy to fabricate. Different from normal FBG, the ellipsoid acts as the cladding mode exciter and coupler, which excites a great amount of cladding modes and recouples them to the fiber core. The SRI can be detected by monitoring the wavelength shift of the cladding resonant peak. In addition, temperature variation will result in a shift for both the Bragg resonant peak and cladding resonant peaks. Moreover, since the displacement of the FBG spectrum with temperature variation is known, the shift of Bragg wavelength can be used as a reliable reference to self-compensate the temperature fluctuation. Therefore, the proposed structure offers a novel refractive index sensor with high sensitivity and self-compensated temperature variation.

## 2. Sensing Principle and Sensor Fabrication

### 2.1. Sensing Principle

**Figure 1:**
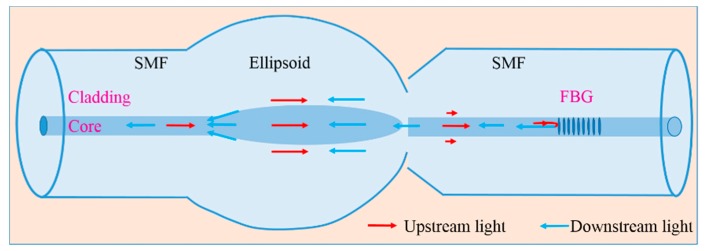
Sensing scheme of the proposed structure. The blue color structure shows the proposed sensor head.

It is well known that standalone FBG can only reflect the core mode, which satisfies the Bragg resonant condition. The total internal reflection determines that the core mode can well be confined in the fiber core, which makes the FBG insensitive to the SRI variation. In contrast, the cladding modes are reflected at the interface between the fiber cladding and the outer medium. Therefore, evanescent waves exist on the surface of the fiber cladding. In this case, the cladding modes can interact with the surrounding medium, and effective indices of the cladding modes can be influenced by the SRI. The basic idea of the proposed structure is to excite cladding modes and recouple the cladding modes into the fiber core in order to have the interference between cladding modes. By monitoring cladding resonant peaks, we can measure SRI. Figure 1 shows the sensing scheme with the proposed structure. The red arrows represent the upstream light, i.e., the input light, and the blue arrows represent the downstream light, i.e., the reflected light. When the input light is injected from the single-mode fiber (SMF) to the ellipsoid, the cladding modes will be excited, and the core mode will be reflected by the FBG. The reflected light will be propagated through the ellipsoid once more, and the cladding modes will be excited and recoupled back to the SMF. The ellipsoid acts as a mode exciter. If without an ellipsoid, a considerable part of the reflected cladding modes will be leaked out after propagating a certain distance.

### 2.2. Sensor Fabrication

**Figure 2:**
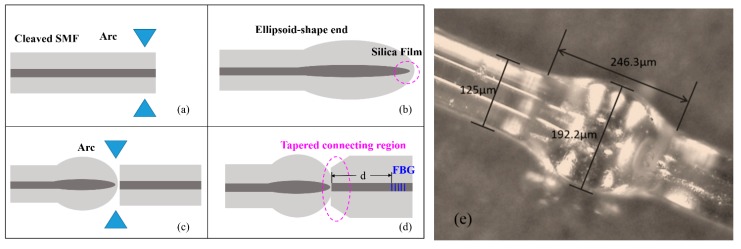
(**a**–**d**) Schematic diagram of the fabrication steps. (**e**) Microscope image of the ellipsoid structure.

According to the fabrication sequence of the ellipsoid and the grating, there are two fabrication schemes of the proposed sensor: (1) an inscription of fiber grating and then fusing with the ellipsoid, and (2) fusing the ellipsoid and then an inscription of fiber grating. The sensor fabricated with the former scheme is easily broken with the valued FBG. Especially, the distance between the ellipsoid and FBG is hard to be controlled precisely. Therefore, the latter scheme is adopted to fabricate the sensor used in this work. The fabrication process is shown in Figure 2a–d. Firstly, a cleaved single mode fiber (Corning, SMF28) was put into the fusion splicer (Fujikura, FSM-100P). The discharge time and intensity were 1300 ms and 200 bits, respectively. After discharging, the fiber tip formed an ellipsoid. The electric arc, which was applied on the surface of the fiber, resulted in the unequable temperature field between the core and the cladding, and thus a silica film at the end of the ellipsoid was formed. Then, the ellipsoid was spliced with an SMF. It is noted that the asymmetrical shape of the ellipsoid and the SMF led to a tapered region. Later on, the sample will be put into the hydrogen loading chamber. After 14 days of hydrogen loading at 10 MPa, the FBG was precisely inscribed in the SMF cascaded with the ellipsoid, as indicated in Figure 2d. The FBG was inscribed using a 248-nm KrF excimer laser (150 Hz, 12 mJ) with a 0.15 mm/s scanning speed. The FBG has a period of 535 nm and a length of 10 mm. Especially, the distance (d) between the ellipsoid and the FBG is precisely controlled from 2 mm to 18 mm for different samples. Figure 2e shows the typical image of the ellipsoid in the proposed sensor, where the length and diameter of the ellipsoid are 246.3 μm and 192.2 μm, respectively.

**Figure 3:**
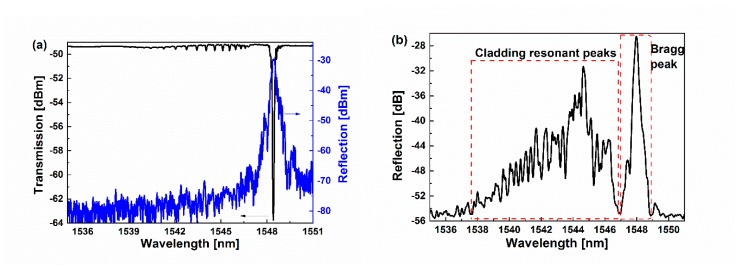
(**a**) Reflection (blue) and transmission (black) spectra of fiber Bragg grating (FBG) cascading the ellipsoid monitored during FBG fabrication with the input direction from the FBG side. (**b**) Reflection spectra of FBG cascading the ellipsoid with the input from the ellipsoid side. The distance d between the ellipsoid and the FBG is 8 mm.

Figure 3a shows the reflection (blue) and transmission (black) spectra of the FBG cascading the ellipsoid monitored by an optical spectrum analyzer (Yokogawa, AQ6370 OSA) during FBG fabrication with the input from the FBG side. Seen from the transmission spectra in Figure 3a, besides the deep Bragg resonant loss peak at 1548.4 nm, there are several ghost loss peaks (cladding resonant peaks) at <1546.5 nm excited by the cascaded ellipsoid. However, none of the characteristic reflections linked with the ghost loss peaks are observed from the corresponding reflection spectrum in Figure 3a. The main reason is that the input light is launched through the FBG side, and there is no chance for the ellipsoid structure to apply the influence upon the reflection spectrum. Figure 3b shows the typical reflection spectrum measured with optical vector analyzer (OVA, Luna, e-4000NF), where the light is launched through the ellipsoid side. Seen from Figure 3b, there is not only one strong Bragg resonant peak at 1545.5 nm, but also a series of cladding resonant peaks from 1537 nm to 1547 nm. The cladding resonant peaks in Figure 3b are linked well with the ghost transmission peaks in Figure 3a, which are all attributed by the excited and recoupled cladding modes by the ellipsoid. In addition, the power difference between the Bragg resonant peak and the maximum cladding resonant peak is only 5 dB as seen in Figure 3b, which indicates that the power of reflected cladding modes is not low and verifies that the ellipsoid is a good cladding mode exciter.

**Figure 4:**
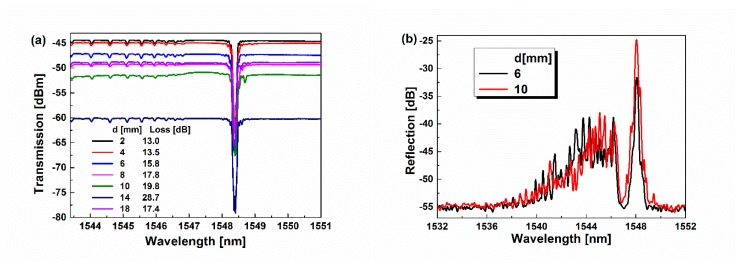
(**a**) Transmission spectra of FBG cascading the ellipsoid with varied distance d. (**b**) Reflection spectra of FBG cascading the ellipsoid with d = 6 mm (black) and 10 mm (red).

To verify the fabrication repeatability of the proposed sensor and the influence of the insertion loss due to the introduction of the ellipsoid, a series of the FBG cascading the ellipsoid with different d have been fabricated with other uniformed conditions. Their transmission spectra are monitored and shown in Figure 4a. Seen from the transmission spectra in Figure 4a, the ghost resonant peaks due to the ellipsoid almost appear at the same wavelength with similar depth, although the distance (d) and loss varied, as listed in the inset of Figure 4a. The insertion loss introduced by the ellipsoid greatly reduces the baseline of the transmission, but it almost has no influence upon the depth and position of ghost resonant peaks. Such observation demonstrates that the proposed structure has low requirements on the fabrication process, which offers the possibility of the fabrication of more compact sensors. Especially, when the ellipsoid is separated from FBG by 6 mm and 10 mm, the reflection spectra of FBG cascading the ellipsoid are measured and shown in Figure 4b. As seen in Figure 4b, the Bragg resonant peak and the cladding resonance peaks almost exist in similar positions in accordance with the observation of the transmission spectra in Figure 4a, although the reflection intensity of these peaks displays some difference, which is possibly due to the fine difference of the ellipsoid shape.

## 3. Sensing Results and Discussion

### 3.1. SRI Sensing

**Figure 5:**
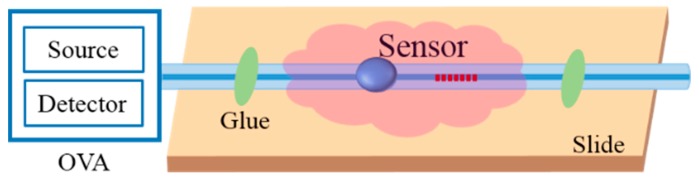
The schematic diagram of the experimental setup.

In order to investigate the SRI sensitivity of the proposed sensor, we carried out a series of experiments to detect the reflection spectra in response to the SRI. The schematic diagram of the experimental setup is shown in Figure 5. The OVA was used to measure the reflection spectra with a standard wavelength resolution of 3.2 pm. Two ends of the sensor head were fixed on a glass slide to keep the fiber stationary during the experiment. A small amount of dimethyl sulfoxide/water solutions with different refractive indices ranging from 1.3352 to 1.4426 were dispensed with a pipette onto the sensor. The refractive indices of the solutions were calibrated by using a commercial Abbe refractometer with the accuracy of 0.0002.

**Figure 6:**
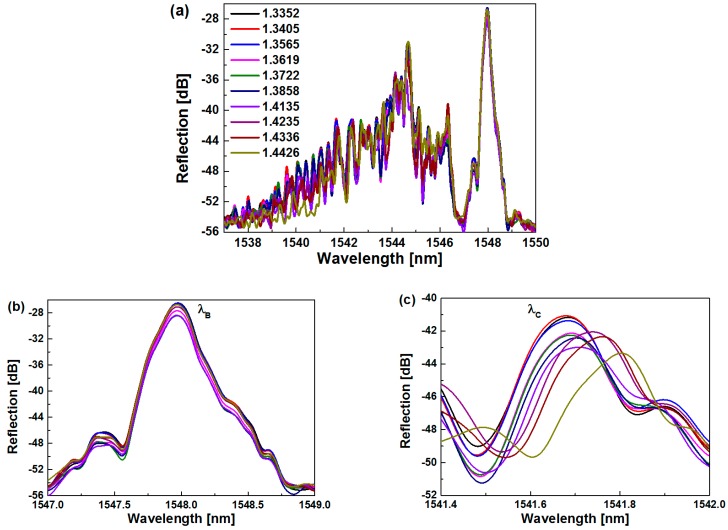
(**a**) Measured spectral responses to different surrounding refractive index (SRI). (**b**) Measured Bragg resonant peak in response to different SRI. (**c**) Measured cladding resonant peak in response to different SRI.

Figure 6a shows the spectral response of the proposed sensor versus the SRI. From Figure 6b,c, it can be seen that as the SRI increases, the Bragg wavelength (*λ_B_*) remains unchanged, but the wavelength of the cladding resonant peak (*λ_C_*) experiences a redshift. The unchanged Bragg wavelength is because the core mode propagates in the FBG, which is not influenced by the SRI. When the modal field of the cladding mode overlaps with the surrounding medium, the effective indices of cladding modes are strongly related to the SRI. As the SRI increases, the effective indices of the cladding modes increase, which has an influence on the coupling between the downstream cladding modes and the upstream core mode. Thus, the redshift of *λ_C_* was observed. After propagating a certain distance, the reflected cladding modes are coupled into the fiber core, and then they will be not influenced by the SRI.

**Figure 7:**
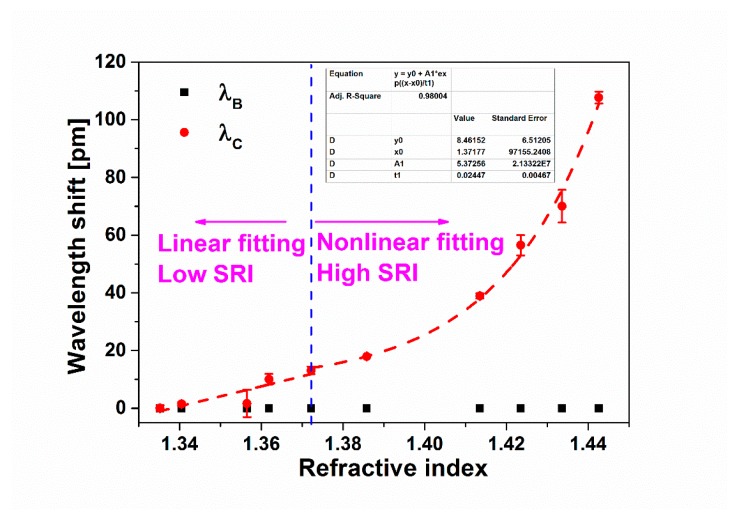
Measured wavelength shifts of cladding and Bragg resonant peaks vs. SRI for the proposed sensor.

Furthermore, Figure 7 shows the shifts of *λ_B_* and *λ_C_* versus the SRI. It can be seen from Figure 7 that as the SRI changes, *λ_B_* remains unchanged, while *λ_C_* moves to the longer wavelength. As shown in the dashed line at the left side, when the SRI increases from 1.3352 to 1.3722 (low SRI), the shift of *λ_C_* has a linear relationship with the SRI, along with a sensitivity of 352.6 pm/RIU. As shown in the dashed line at the right side, when the SRI increases from 1.3722 to 1.4426 (high SRI), the fitting curve follows an essentially exponential function, along with a maximum sensitivity of 4182.2 pm/RIU from 1.4336 to 1.4426. The obtained sensitivity is not higher than some previous reports [23,24,25,26,30], and the sensitization technique is desired to further improve the SRI sensitivity. The relationship between the wavelength shift Δ*λ_C_* and SRI can be described as following:

(1) $$\Delta \lambda_{C}=f\left(n\right)=\{\begin{array}{lll} 352.565\times \left(n-1.3352\right) & & 1.3352\leq n\leq 1.3722\\ 8.462+5.373\times e^{\frac{n-1.37177}{0.02447}} & & 1.3722<n\leq 1.4426 \end{array}$$

where the transition between the linear and non-linear fittings is set at *n* = 1.3722. If the fitting transition is set at *n* = 1.3858, the fitting adjusted R^2^ for the non-linear region will be reduced from 0.98 to 0.96. In addition, it is noted that more data points for *n* = 1.38–1.41 will be greatly beneficial for the consolidation of the select of the fitting transition. From Figure 7 and Equation (1), two separate fittings are obtained. The main reason is considered as follows. The evanescent waves rapidly weaken to vanish at the boundary of fiber when the SRI is much lower than the refractive index of the fiber cladding. However, as SRI increases to be larger than 1.4, the restriction effect of the fiber boundary reduces, and the evanescent waves propagate farther, resulting in a higher SRI sensitivity. Especially, when this refractive index of the ambient medium is very close to the refractive index of silica, the cladding modes will not be guided by the fiber well, since the boundary between two media disappears, finally resulting in almost ~10 times higher maximum sensitivity compared with a low SRI range [38].

### 3.2. Temperature Response

**Figure 8:**
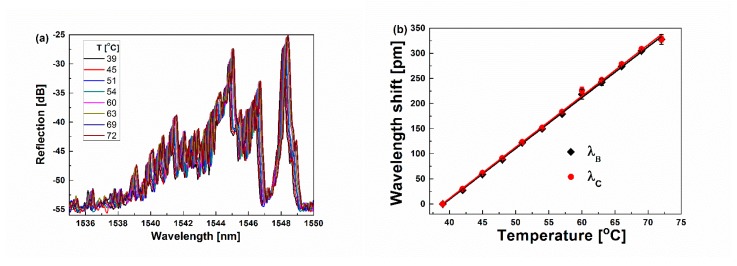
(**a**) Reflection spectra of the proposed sensor (d = 4 mm) at different temperatures. (**b**) Measured wavelength shift vs. temperature for the FBG and ellipsoid structure.

When the proposed sensor is used for SRI sensing, it is inevitable to encounter the temperature fluctuation. So, it is necessary to investigate the temperature response of the proposed sensor to minimize or eliminate the cross influence from the temperature variation in the SRI sensing. Here, we carried out a series of temperature tests by immersing the sensor at the bottom of the water tank (~20 L). For ease of operation, the hot water was poured into the water tank. With the water cooling down naturally (the actual temperature of the water was measured by the thermometer), the reflection spectra of the sensor were monitored in situ with OVA. Figure 8a shows the reflection spectra at different temperatures. As seen in Figure 8a, with the temperature increasing, the reflection spectra redshift, and the reflection intensities increase due to the minor decrement of the refractive index of water. However, the shape of the reflection profiles is almost kept the same. Furthermore, Figure 8b shows the dependences of the shift of *λ_B_* and *λ_C_* on the temperature change. It can be seen that both *λ_B_* and *λ_C_* linearly shift about 328.0 pm when the temperature changes from 39 °C to 72 °C, along with almost the same temperature sensitivity (*K_TB_* = *K_TC_*) of ~10.76 pm/°C.

Actually, the shift of *λ_B_* and *λ_C_* should have some difference, as the cladding resonant peaks are affected by the change from both temperature and refractive index of water (due to the thermo-optic effect of the water), while the Bragg resonant peak is only affected by the temperature change. According to the reference [39], the refractive index of water almost reduces linearly from 1.3199 to 1.3142 with the temperature increasing from 39 to 72 °C. Here, it is assumed that the shift of *λ_C_* should follow Equation (1) in the linear range (low SRI). In such case, *λ_C_* shifts due to the variation of the refractive index by the temperature change from 39 to 72 °C being approximately −2.0 pm (given out a temperature response of approximately 0.06 pm/°C), which is about 0.6% of the wavelength shift, as shown in Figure 8b. It is not only lower than the standard wavelength resolution of OVA (3.2 pm), but also much lower than the wavelength shift (328.0 pm) that resulted from the temperature change in Figure 8b. Therefore, the contribution of SRI change from the temperature variation upon Δ*λ_C_* can almost be neglected, resulting in the same temperature sensitivity of ~10.76 pm/°C for both *λ_B_* and *λ_C_*. Such a result also demonstrates that the temperature sensitivity of the sensor is mainly determined by the thermo-optic effect and thermal expansion effect of silica fiber, similar to the normal FBG sensor.

### 3.3. Sensing Response with Different Sensors

**Figure 9:**
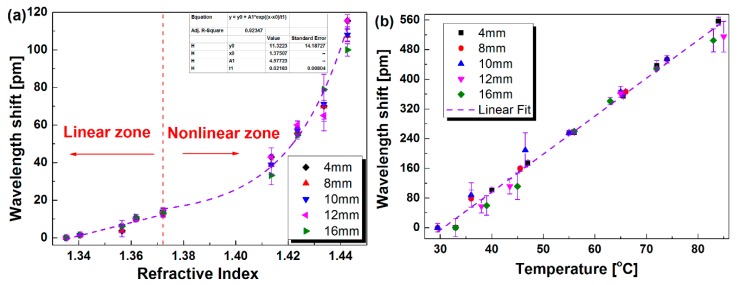
Measured wavelength shift of cladding resonant peak vs. SRI (**a**) and temperature (**b**) for the proposed sensor with different d.

As shown in Figure 4b, with different d values, the cladding resonant peaks of the proposed sensor are located in a similar wavelength range, although their reflection intensities are different from each other. So, it is interesting to see if there is an optimum d, giving out the higher sensitivity. Here, we carried out the experiment of SRI sensing and temperature response with the proposed sensors with varied d values. Similar to the results in Figure 6 and Figure 8, the Bragg wavelength almost has no change with the SRI variation, while it displays a similar response to the temperature change as that of the cladding resonant peak. Especially, the wavelength shift of cladding resonant peak versus SRI and temperature are measured and plotted in Figure 9a,b, respectively. As seen in Figure 9a, *λ_C_* moves to the longer wavelength as the SRI increases. Similarly, when the SRI is lower than 1.3722, it almost increases linearly with the increment of the SRI, giving out a similar average sensitivity of 352.6 pm/RIU. In addition, when the SRI is over 1.3722, it also follows with the exponential growth, which fits with the function in the inset of Figure 9a. As seen from Figure 9b, *λ_C_* also linearly redshifts as the temperature increases, along with an average temperature sensitivity of approximately 10.23 pm/°C. These results in Figure 9 indicate that the wavelength shift of the cladding resonant peak responding to both SRI and temperature almost has no relationship with the distance d, which further demonstrates the robust performance of the proposed sensor and the ease of the fabrication.

### 3.4. Repeatability Test

**Figure 10:**
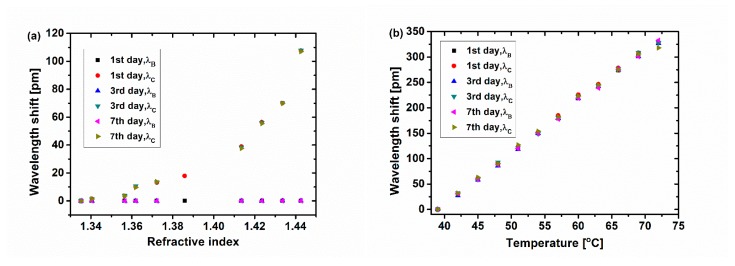
(**a**) SRI repeatability test on different days. Note: the result of *n* = 1.3858 was obtained only on the first day, as the index solution was damaged after the first day’s experiment. (**b**) Temperature repeatability test on different days.

Repeatability is a key property for the practical application of a sensor. In order to analyze the repeatability of the proposed sensor, several measurements of SRI and temperature have been carried out by using the same sensor and under the same situation but on different days, as shown in Figure 10a,b, respectively. Figure 10a displays the experimental results of SRI detection repeatability on the first day, third day, and seventh day. It can be seen that cladding and Bragg resonant peaks almost have the same wavelength shift on different days. The biggest offset of the wavelength shift is 0.6 pm when the SRI is 1.3722 on the first day and seventh day, which is only 4.5% of the total wavelength shift. The experimental results of temperature test repeatability on different days can be seen in Figure 10b. The wavelength shift of the cladding and Bragg resonant peaks is almost the same. The maximum deviation value is 9.0 pm, which is only 2.7% of the total wavelength shift when the temperature is 72 °C; this deviation is probably caused by an inaccurate reading of the thermometer. So, it can be considered that the proposed sensor with FBG cascading the ellipsoid has good repeatability of SRI and temperature response for potential application.

### 3.5. Temperature Self-Compensated SRI Sensing

As mentioned in Section 3.1, the Bragg wavelength (*λ_B_*) is almost insensitive to the SRI change, while the relation between the wavelength shift of the cladding resonant peak (Δ*λ_C_*) and the SRI follows Equation (1). In addition, the experimental data in Section 3.2 indicate that the response of *λ_B_* and *λ_C_* to the temperature variation (Δ*T*) is almost linear, giving out the same temperature sensitivity (*K_TB_* = *K_TC_*) of approximately 10.76 pm/°C and the relation as below:

(2) $$\Delta \lambda_{B}=K_{TB}\Delta T=K_{TC}\Delta T$$

when immersing the proposed sensor into the solution to monitor the change of the SRI with temperature fluctuation, we can remove the influence of temperature change (Δ*T*) upon the shift of the cladding resonant peak (Δ*λ_C_*) by subtracting the shift of *λ_B_* (Δ*λ_B_*) as below,

(3) $$\Delta \lambda_{C}=f\left(n\right)+K_{TC}\Delta T=f\left(n\right)+K_{TB}\Delta T=f\left(n\right)+\Delta \lambda_{B}$$

realizing a temperature self-compensated SRI sensor. Given the wavelength resolution of the optical vector analyzer of 3.2 pm, according to Equation (3), the resolution in terms of SRI sensing is up to 0.0091 (calculated with the sensitivity of 352.6 pm/RIU).

**Figure 11:**
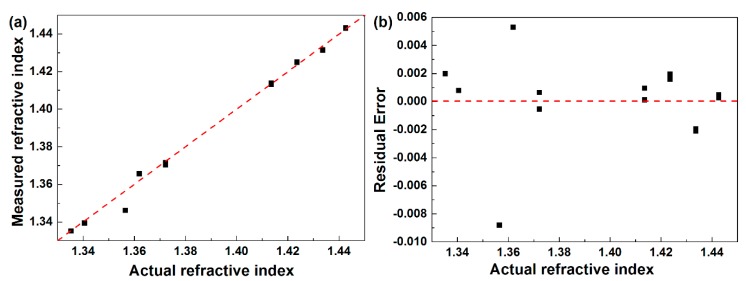
(**a**) Measured refractive index vs. actual refractive index. (**b**) Measured error for each known SRI.

Furthermore, based on the proposed sensor, the measurements of a series of known SRI solutions have been performed. According to Equations (1)–(3), the refractive indices of these known SRI values were measured, where the measured SRI values versus the actual SRI values are plotted in Figure 11a. As seen from Figure 11a, most of the points are close to the red dash line, which further verified the proposed sensor. Especially, the measurement error was plotted in Figure 11b. As seen from Figure 11b, the measured error is ranged from −0.0088 to 0.0053, giving out an averaged measured error of 0.0037. All these results indicate that the proposed structure works well as a temperature self-compensated refractive index sensor.

## 4. Conclusions

In summary, we report a temperature self-compensated refractive index sensor with large SRI values ranging from 1.3352 to 1.4426 based on FBG cascading an ellipsoid. The proposed sensor utilizes the ellipsoid to excite and recouple the cladding modes into the fiber core. The SRI can be determined by measuring the wavelength shift of the cladding resonant peak, and a large detection range from 1.3352 to 1.4426 and a maximum sensitivity of 4182.2 pm/RIU can be achieved. The cladding resonant peak has the same temperature sensitivity with that of the Bragg resonant peak, and thus the temperature fluctuation can be self-compensated when the sensor is used for the SRI sensing with the temperature variation. Especially, the sensing performance has no special requirement, so it enables an ease of fabrication. It is believed that our work can serve as a prerequisite for the further development of other SRI sensors based on FBG cascading the ellipsoid, providing the introduction to many sensitization techniques.

## References

[1] Lu P., Men L., Sooley K., Chen Q. (2009). Tapered fiber Mach-Zehnder interferometer for simultaneous measurement of refractive index and temperature. Appl. Phy. Lett..

[2] Sun L., Semenova Y., Wu Q., Liu D., Yuan J., Ma T., Sang X., Yan B., Wang K., Yu C. (2017). High sensitivity ammonia gas sensor based on a silica gel coated microfiber coupler. J. Lightwave Technol..

[3] Sun L., Yuan J., Ma T., Sang X., Yan B., Wang K., Yu C. (2017). Design and optimization of silicon concentric dual-microring resonators for refractive index sensing. Opt. Commun..

[4] Bo L., O’Mahony C.C., Semenova Y., Gilmartin N., Wang P., Farrell G. (2014). Microfiber coupler based label-free immunosensor. Opt. Express.

[5] Wu Q., Semenova Y., Wang P., Farrell G. (2011). High sensitivity SMS fiber structure based refractometer–analysis and experiment. Opt. Express.

[6] Chen C., Yang R., Zhang X., Wei W., Guo Q. (2018). Compact refractive index sensor based on an S-tapered fiber probe. Opt. Mater. Express.

[7] Fan Z. (2019). A tunable high-sensitivity refractive index of analyte biosensor based on metal-nanoscale covered photonic crystal fiber with surface plasmon resonance. IEEE Photonics J..

[8] Chan C.F., Chen C., Jafari A., Laronche A., Thomson D.J., Albert J. (2007). Optical fiber refractometer using narrowband cladding-mode resonance shifts. Appl. Opt..

[9] Li X.H., Zhang Z., Qin S., Wang T., Liu F., Qin M., Su Y. (2009). Sensitive label-free and compact biosensor based on concentric silicon-on-insulator microring resonators. Appl. Opt..

[10] Yi H., Citrin D.S., Zhou Z. (2010). Highly sensitive silicon microring sensor with sharp asymmetrical resonance. Opt. Express.

[11] Boyd R.W., Heebner J.E. (2001). Sensitive disk resonator photonic biosensor. Appl. Opt..

[12] Kersey A.D., Davis M.A., Patrick H.J., LeBlanc M., Koo K.P., Askins C.G., Putnam M.A., Friebele E.J. (1997). Fiber grating sensors. J. Lightwave Technol..

[13] Barmenkov Y.O., Ortigosa-Blanch A., Diez A., Cruz J.L., Andrés M.V. (2004). Time-domain fiber laser hydrogen sensor. Opt. Lett..

[14] Kuzyk M.G. (2006). Polymer Fiber Optics: Materials, Physics, and Applications.

[15] Arellano-Sotelo H., Kir’yanov A.V., Barmenkov Y.O., Aboites V. (2011). The use of nonlinear dynamics of erbium-doped fiber laser at pump modulation for intra-cavity sensing. Opt. Laser Technol..

[16] Martin-Vela J.A., Sierra-Hernandez J.M., Martinez-Rios A., Estudillo-Ayala J.M., Gallegos-Arellano E., Toral-Acosta D., Porraz-Culebro T.E., Jauregui-Vazquez D. (2019). Curvature sensing setup based on a fiber laser and a long-period fiber grating. IEEE Photonics Technol. Lett..

[17] Rao Y.J. (1997). In-fibre Bragg grating sensors. Meas. Sci. Technol..

[18] Guo T., Tam H.Y., Krug P.A., Albert J. (2009). Reflective tilted fiber Bragg grating refractometer based on strong cladding to core recoupling. Opt. Express.

[19] Sáez-Rodriguez D., Cruz J.L., Díez A., Andrés M.V. (2011). Coupling between counterpropagating cladding modes in fiber Bragg gratings. Opt. Lett..

[20] Wu Q., Okabe Y., Wo J. (2015). Fiber sensor based on interferometer and Bragg grating for multiparameter detection. IEEE Photonics Technol. Lett..

[21] Zhu T., Wu D., Liu M., Duan D.W. (2012). In-line fiber optic interferometric sensors in single-mode fibers. Sensors.

[22] Yuan S., Tong Z., Zhao J., Zhang W., Cao Y. (2014). High temperature fiber sensor based on spherical-shape structures with high sensitivity. Opt. Commun..

[23] Bo L., Wang P., Semenova Y., Farrell G. (2013). High sensitivity fiber refractometer based on an optical microfiber coupler. IEEE Photonics Technol. Lett..

[24] Xu F., Brambilla G., Lu Y. (2009). A microfluidic refractometric sensor based on gratings in optical fibre microwires. Opt. Express.

[25] Lou J., Wang Y., Tong L. (2014). Microfiber optical sensors: A review. Sensors.

[26] Li L., Xia L., Xie Z., Liu D. (2012). All-fiber Mach-Zehnder interferometers for sensing applications. Opt. Express.

[27] Caucheteur C., Guo T., Albert J. (2017). Polarization-assisted fiber Bragg grating sensors: Tutorial and review. J. Lightwave Technol..

[28] Zhang A.P., Tam H.Y., Tao X.M. (2003). Mode recoupling in a novel Bragg grating pair. Opt. Lett..

[29] Shu X., Gwandu B.A.L., Liu Y., Zhang L., Bennion I. (2001). Sampled fiber Bragg grating for simultaneous refractive-index and temperature measurement. Opt. Lett..

[30] Chen N., Yun B., Cui Y. (2006). Cladding mode resonances of etch-eroded fiber Bragg grating for ambient refractive index sensing. Appl. Phys. Lett..

[31] Chen X., Zhou K., Zhang L., Bennion I. (2005). Simultaneous measurement of temperature and external refractive index by use of a hybrid grating in D fiber with enhanced sensitivity by HF etching. Appl. Opt..

[32] Ling Q., Gu Z., Gao K. (2018). Smart design of a long-period fiber grating refractive index sensor based on dual-peak resonance near the phase-matching turning point. Appl. Opt..

[33] Esposito F., Ranjan R., Campopiano S., Iadicicco A. (2017). Experimental study of the refractive index sensitivity in arc-induced long period gratings. IEEE Photon. J..

[34] Coelho L., Viegas D., Santos J.L., de Almeida J.M.M.M. (2016). Characterization of zinc oxide coated optical fiber long period gratings with improved refractive index sensing properties. Sens. Actuator. B.

[35] Chamorro Enríquez D.A., da Cruz A.R., Rocco Giraldi M.T.M. (2012). Hybrid FBG–LPG sensor for surrounding refractive index and temperature simultaneous discrimination. Opt. Laser Technol..

[36] Zhou B., Zhang A.P., He S., Gu B. (2010). Cladding-mode-recoupling-based tilted fiber Bragg grating sensor with a core-diameter-mismatched fiber section. IEEE Photonics J..

[37] Liu X., Zheng J., Yang J., Li Y., Dong X. (2015). Refractive index sensor based on combination of tilted fiber Bragg grating and waist-enlarged fusion bitaper. Opt. Commun..

[38] Ivanov O.V., Zlodeev I.V. (2013). Fiber structure based on a depressed inner cladding fiber for bend, refractive index and temperature sensing. Meas. Sci. Technol..

[39] Bashkatov A.N., Genina E.A. Water refractive index in dependence on temperature and wavelength: A simple approximation. Proceedings of the Saratov Fall Meeting 2002: Optical Technologies in Biophysics and Medicine IV.

